# Short Flax Fibres and Shives as Reinforcements in Bio Composites: A Numerical and Experimental Study on the Mechanical Properties

**DOI:** 10.3390/polym15102239

**Published:** 2023-05-09

**Authors:** Sofie Verstraete, Bart Buffel, Dharmjeet Madhav, Stijn Debruyne, Frederik Desplentere

**Affiliations:** 1 Research Group ProPoliS, Department of Materials Engineering, KU Leuven Campus Bruges, Spoorwegstraat 12, 8200 Bruges, Belgium; 2 Surface and Interface Engineered Materials, Department of Materials Engineering, KU Leuven Campus Bruges, Spoorwegstraat 12, 8200 Bruges, Belgium; 3 Research Group M-Group, Department of Mechanical Engineering, KU Leuven Campus Bruges, Spoorwegstraat 12, 8200 Bruges, Belgium

**Keywords:** flax fibres, injection moulding, mechanical properties, short fibre reinforced thermoplastics (SFRT), bio composites

## Abstract

The complete flax stem, which contains shives and technical fibres, has the potential to reduce the cost, energy consumption and environmental impacts of the composite production process if used directly as reinforcement in a polymer matrix. Earlier studies have utilised flax stem as reinforcement in non-bio-based and non-biodegradable matrices not completely exploiting the bio-sourced and biodegradable nature of flax. We investigated the potential of using flax stem as reinforcement in a polylactic acid (PLA) matrix to produce a lightweight, fully bio-based composite with improved mechanical properties. Furthermore, we developed a mathematical approach to predict the material stiffness of the full composite part produced by the injection moulding process, considering a three-phase micromechanical model, where the effects of local orientations are accounted. Injection moulded plates with a flax content of up to 20 V% were fabricated to study the effect of flax shives and full straw flax on the mechanical properties of the material. A 62% increase in longitudinal stiffness was obtained, resulting in a 10% higher specific stiffness, compared to a short glass fibre-reinforced reference composite. Moreover, the anisotropy ratio of the flax-reinforced composite was 21% lower, compared to the short glass fibre material. This lower anisotropy ratio is attributed to the presence of the flax shives. Considering the fibre orientation in the injection moulded plates predicted with Moldflow simulations, a high agreement between experimental and predicted stiffness data was obtained. The use of flax stems as polymer reinforcement provides an alternative to the use of short technical fibres that require intensive extraction and purification steps and are known to be cumbersome to feed to the compounder.

## 1. Introduction

To address the end-of-life challenges of thermoset composite materials, the use of thermoplastic as matrix material is receiving more and more attention in academia and industry. Thermoplastic matrices allow faster cycle times and are applicable in large volume processes such as extrusion, compression moulding or injection moulding. The latter two processes allow the production of complex parts at relatively low production costs. However, these production processes restrict the reinforcement to short fibres. Short fibre-reinforced thermoplastics are therefore used in applications where ultimate strength or stiffness are not the main requirements [1].

As the awareness for sustainable resources is growing, recent trends show an increasing interest in the use of natural fibres as a bio-based alternative to synthetic fibres [2,3,4,5,6]. Flax fibres with mechanical properties that even outperform the specific stiffness of glass fibres are among the most commonly used natural fibre reinforcements [7]. The cultivation of flax fibres is characterised by a low environmental impact and relatively short sowing-to-harvesting cycle of approximately 100 days. Cultivation factors such as flax species, geographical location, nutrition, temperature, season and local climate conditions affect the mechanical properties and overall quality of natural fibres [8,9]. To cope with this inevitable variation in properties, special regulations and monitoring of cultivation are applied. This ensures the high quality of flax and reduces the variation in mechanical properties [10]. After harvesting, the flax stems are carefully and evenly spread on the soil to undergo a dew-retting process. The flax fibres and the wooden parts, also called shives, are hereby separated by a natural process of pectin degradation that binds the fibres with shives [11]. After retting, flax stem is broken, and the shives are separated from the fibres by a scutching process. Finally, flax fibres are hackled to detangle and straighten producing individual technical fibres that are suited for use in the textile industry or long-fibre reinforced composites [12,13].

Further advances towards “green composites” require the development and utilisation of alternative bio-based polymers. These polymers are synthesised out of renewable feedstock such as sugar, starch, cellulose, lignin and show an interesting combination of environmental friendliness and mechanical properties [14]. A starch-based polylactic acid (PLA) biopolymer can achieve Young’s modulus of 3 GPa, tensile modulus of up to 70 MPa and impact strength of 2.5–3 kJ/m^2^ [15]. The literature indicates that adding short flax fibres to a PLA matrix preserves the possibility of using the material in an injection moulding process, while the tensile modulus increases up to 5 GPa at a volume fraction of 20% [16,17].

Shives are separated from the flax stem during the production process of flax fibres and represent agricultural waste that is currently used as animal bedding, particleboard, thermal insulation material for buildings [18] or even as an alternative to the widely used wood flour plastic composites (WPC) [19]. About 70 to 85% (depending on the flax variety) of the flax stem consists of shives [20]. As France, Belgium and the Netherlands combined have a total flax cultivation area of over 124,000 ha, it is estimated that about 400,000 tonnes of flax shives was available in 2018. This amount is expected to further increase due to the growing interest in the application of natural fibres [21].

Shives were found to be responsible for 30% of the bending stiffness of the dry flax stem [20]. Nuez et al. investigated the potential of these flax shives as reinforcement in a polymetric matrix and found an improvement in the mechanical properties of a flax shive/polypropylene composite [22]. In another study by Soete et al., the fibre breakage of the flax stem during an injection moulding process was investigated [23]. They showed that the high shear forces during the compounding and injection moulding process are responsible for fibre breakage, resulting in rupture of the flax fibres and the wooden parts or flax shives that are adjacent to each other in the composite. Results of the tensile tests showed an improvement in mechanical properties when used in a PP matrix [24]. Results of the tensile tests on hackled flax fibre-reinforced PP showed an improvement in mechanical properties [24].

Since the use of flax shives as well as the whole flax stem as reinforcements in a PP matrix have shown promising results, there is a potential for high-value application of this agricultural waste product. Therefore, the present study focuses on the use of flax shives as well as the whole stem in a bio-based PLA matrix. The obtained composite is then 100% bio-based and biodegradable. By using the whole stem of the flax plant as reinforcement, energy-consuming production steps to extract the technical fibres are avoided. This leads to a reduction in the production cost of the material while maintaining the interesting mechanical properties of the composites.

The effect of the reinforcements strongly depend on the size of the fibres. In the case of continuous fibre-reinforced composites, application of nano particles in a polymetric matrix result in an increase in mechanical (tensile, flexural, fatigue, …) properties or flame retardant improvement [25,26,27,28,29,30], or they can act to improve the mechanical properties for long fibre composites [31]. For short fibre composites, an optimal design of the fibre orientation extends the life of the composite system while lowering production cost and minimising the fibre volumetric percentage [32]. When a short fibre composite is produced through injection moulding, it is well documented that the fibre orientation is determined by the flow of the material. In this way, the typical skin–shell–core orientation is obtained [1]. The orientation of the short fibres mainly depends on the rheological properties of the molten compound, which is directly affected by the aspect ratio and volume fraction of fibre in the compound. This results in non-isotropic and non-orthotropic mechanical properties of the composite. The ratio of the minimum and maximum value of tensile modulus is defined as the degree of anisotropy in the material, which increases with increasing fibre volume fraction [33]. The literature reports a higher tensile modulus and strength along the flow direction of injection moulded parts [34,35]. The coupling of the injection moulding production process and the obtained mechanical properties is inevitable and must be considered during the design phase of a part. When used in larger assemblies for semi-structural applications, considering the local anisotropic material properties is mandatory [36].

Therefore, in the present study, the stiffness ratio along the two orthogonal directions was considered to evaluate the degree of anisotropy, and a three-phase [37] multi-layer analytical model was established to predict the mechanical stiffness along all orientations of an injection moulded plate. The approach is based on the Mori–Tanaka (MT) micromechanics model combined with general laminate theory (GL) and considers the aspect ratio, mechanical properties and process-induced orientations of the flax fibres and shives.

## 2. Materials and Methods

### 2.1. Materials

The thermoplastic matrix material considered in this study is a commercially available polylactic-acid (PLA) 4043D Ingeo biopolymer (manufactured by NatureWorks, Minneapolis, MN, USA) with a melt flow index (MFI) of 6 g/10 min (210 °C, 2.16 kg) and a density of 1.24 g/cm^3^.

Flax straw (consisting of technical flax fibres and flax shives) and flax shives were provided by ABV (Algemeen Belgisch Vlasbond, Kortrijk, Belgium) and used as reinforcement to the PLA material. Short E-glass fibres are used as reference material to benchmark the performance of the flax reinforcements. The initial properties of the considered fibres are summarised in Table 1.

### 2.2. Production Process of the Bio-Based Composite

The composites are produced using a two-step production process. First, a compounding step is used to blend the reinforcement into the PLA matrix. Subsequently, samples are produced through injection moulding. Prior to each processing step, the PLA matrix and the flax reinforcements are dried with a Moretto pressurised air dryer at 80°C for 6 h. A Leistritz ZSE18MAXX (Nürnberg, Germany) corotating twin-screw laboratory extruder was used to shear-blend the components and produce pellets with a length of 5 mm. The details of the compounding step are shown in Table 2. Compounds of PLA with up to 15 V% flax shives, up to 20 V% straw flax (flax shives + flax fibres) and up to 20 V% glass fibres were prepared. In the following sections, the volume fraction is referred to as the volume percentage and is therefore indicated with the unit %. The compounding screw elements were selected to avoid excessive shear forces to reduce fibre breakage.

**Figure 1:**
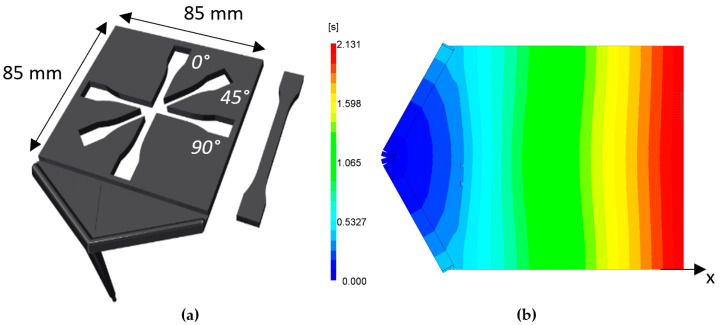
Plate mould geometry showing (**a**) the production of tensile bars (ISO 527-1BA) under different orientations for material characterisation and (**b**) the filling time of the plate, indicating a parallel flowing front during injection moulding process.

After the second drying step of the compound (80 °C, 6 h), a batch of 15 plates of 85 mm × 85 mm × 3 mm was produced using an Arburg 320S injection moulding machine (Loßburg, Germany). The studied compounds exist of virgin PLA, PLA with 5-, 10- and 15% flax shives (FS), 5-, 10- and 20% straw flax fibres (FF) and 10- and 20% of glass fibres (GF). Tensile bar samples are milled out of the injection moulded plates at different orientations with respect to the flow direction of the melt. These tensile bars are used for mechanical testing (Figure 1a). The parameters of the injection moulding process are summarised in Table 3.

A fan-gate injection sprue is used to obtain a parallel flow front in the plate, as shown in Figure 1b. This parallel flow front ensures a high fibre orientation along the flow direction, which allows for the investigation of the tensile modulus of composites along different orientations with respect to the flow direction.

### 2.3. Fibre Morphology

During twin screw compounding, high shear forces are exerted by the screw onto the material. These shear forces separate the technical fibres from the shives, but also inevitably cause unwanted fibre breakage and damage [24]. At each step of the production process, the morphology and dimensions of the fibres and shives were determined using an extraction process. This comprises dissolving the PLA material in chloroform for 24 h. The shives were separated from the fibres based on density, and the chloroform was removed from each component by filtration.

The straw flax structure was examined by optical laser microscopy. The fibre was embedded in an epoxy resin (EpoxyFix for embedding materialographic specimens, Struers, Kopenhagen, Denmark), and the surface was polished using a diamond paste with grain size of 1 µm. The composition was determined by weighting the two components from at least 3 batches and at different volume fractions, as described by [23]. Dimensions of the reinforcements, such as aspect ratio (AR) and volume fraction, were measured using a reflection mode optical laser microscope (VK1050, Keyence, Mechelen, Belgium) in combination with an image processing algorithm in MATLAB.

### 2.4. Mechanical Characterisation

ISO 527-1BA dog bone specimens were produced, using a milling process, at different orientations relative to the flow direction (0°, 30°, 45°, 60° and 90°) from the plates (PLA, PLA + 5-, 10- and 15% FS, PLA + 5-, 10-, 20% FF and PLA + 10- and 20% GF). Three specimens of each compound were tested at room temperature using an Instron 3367 two-column tensile bench equipped with a 30 kN load cell (Darmstadt, Germany), in combination with an extension meter (Instron 2630–125). A crosshead speed of 1.3 mm/min was used. Additional tests were performed in combination with a digital image correlation setup (stereo-DIC, Limess, Krefeld, Germany) to obtain values for Poisson’s ratio. The CMOS cameras with a resolution of 2.3 Mpx were calibrated using an A6 (type: KL 50108) calibration grid. Results were measured with a frequency of 4 Hz and analysed in the ISTRA4D (4.6) software. A summary of the setup is given in Table 4, as described in [39].

## 3. Theoretical Analysis

A numerical method is developed to determine the elastic properties of straw flax-reinforced thermoplastics. The result of this numerical method is compared to experimental data.

### 3.1. Three-Phase Mori–Tanaka (MT) Modelling

**Figure 2:**
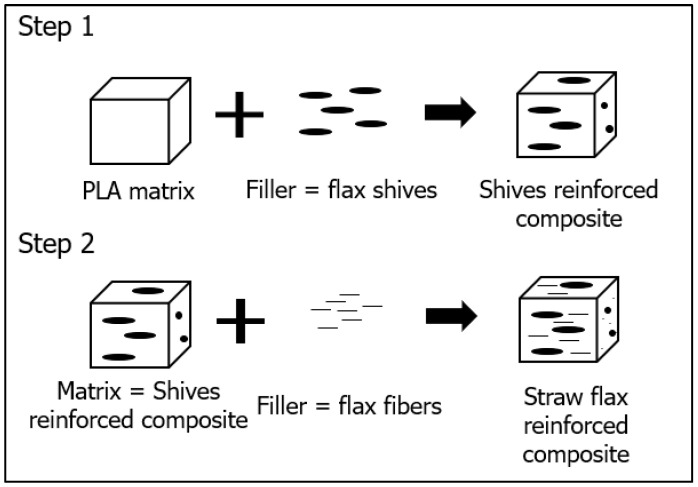
Setup of a Mori–Tanaka model for a three-phase system.

The Mori–Tanaka micromechanics approach is selected to determine the elastic properties. Since straw flax consists of flax fibres and shives, the composite is considered to be a three-phase system [40]. The different phases are illustrated in Figure 2. The first step considers the composite made of neat PLA and flax shives. In the second step, this composite is reinforced with flax fibres. In each step, the individual elastic properties and dimensions of both reinforcing components are taken into account.

The stiffness tensor of the flax straw reinforced composite is calculated by the generalised Mori–Tanaka homogenisation procedure [35]. In the first step of this three-phase system, the stiffness tensor $C_{comp\_1}$ is described by a Mori–Tanaka homogenisation model presented in Equation (1). This compliance refers to the neat PLA reinforced with flax shives. The second homogenisation step, described in Equation (2), calculates the stiffness matrix $C_{comp}$ for the full straw flax composite. In this step, the shives-reinforced composite is considered to be the matrix material and the flax fibres the reinforcement.

(1) $$C_{comp\_1}=C_{m}+V_{s}\left(C_{s}-C_{m}\right)A^{Mori-Tanaka\_1}$$

(2) $$C_{comp}=C_{comp\_1}+V_{f}\left(C_{f}-C_{comp\_1}\right)A^{Mori-Tanaka\_2}$$

The subscript m represents the PLA matrix, s the flax shives and f the flax fibres. V indicates the volume fraction corresponding to the relevant composition of the fibres or shives, and $A^{Mori-tanaka}$ is the concentration strain, as defined by Mori–Tanaka [35],

(3) $$A^{Mori-Tanaka\_x}=A{\left[\left(1-V_{f}\right)I+V_{f}A\right]}^{-1}$$

(4) $$A\;={\left[I\;+\;S\left(C_{m}^{-1}\right)\left(C_{f}-{\;C}_{m}\right)\right]}^{-1}$$

where S represent the Eshelby strain tensor. This tensor is based on the assumption of an elliptic inclusion in which the width and height of the inclusion are assumed to be equal ($a_{2}=a_{3})$ [35]. The aspect ratio of the inclusion is calculated as ρ=a1a2, where the values for $a_{1}$ and $a_{2}$ are experimentally determined values for shives and technical fibres.

### 3.2. Orientation

**Figure 3:**
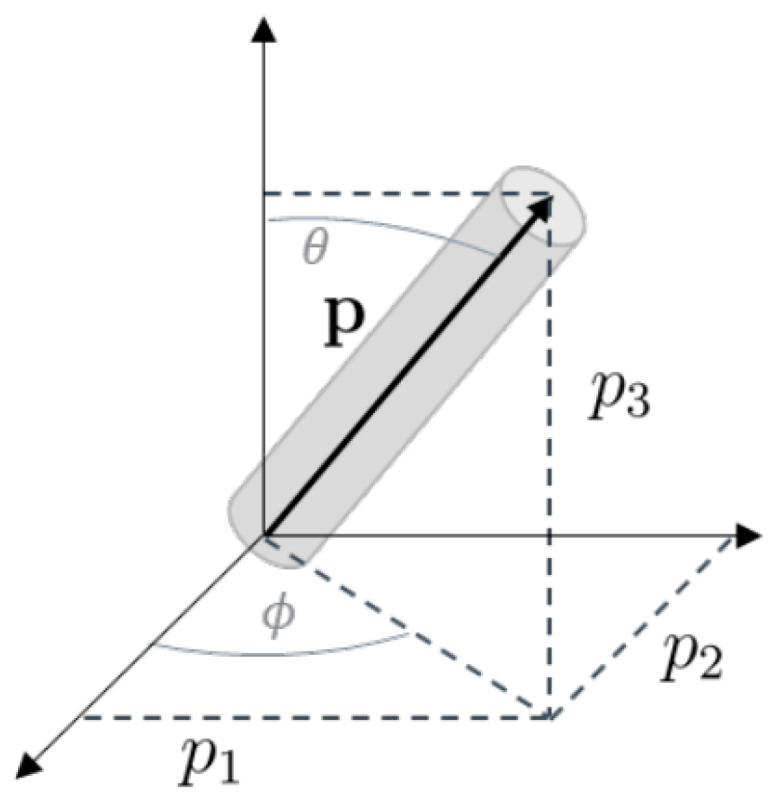
Coordinate system of unit vector p.

Subsequently, the effect of the fibre orientation on the elastic properties is evaluated. During injection moulding, the short fibres are immersed in a highly viscous polymer melt. The final orientation distribution of the fibres is determined by the rheological behaviour of the compound and by the injection moulding process conditions. The orientation of the fibres is described by the probability distribution function $\Psi \left(p\right)$. In this function, the orientation of the fibres is associated with unit vector p. The components of p are related to the angles θ and Φ, as shown in Figure 3 [40].

(5) $$p_{1}=sin\theta \;cos\Phi$$

(6) $$p_{2}=sin\theta \;sin\Phi$$

(7) $$p_{3}=cos\theta \;$$

.

A set of second-order orientation tensors is defined by forming dyadic products of the vector p and integrating them with the distribution function over all possible directions, as given in Equation (8). Each element of the orientation tensor is described as $a_{ij}=\;\oint p_{i}p_{j}\Psi \left(p\right)dp$ [40]. The subscripts i and j represent values between 1 and 3, referring to the orientations in Equations (5)–(7).

(8) $$P=\left[\begin{array}{ccc} \left\langle p_{1}p_{1}\right\rangle & \left\langle p_{1}p_{2}\right\rangle & \left\langle p_{1}p_{3}\right\rangle \\ \left\langle p_{2}p_{1}\right\rangle & \left\langle p_{2}p_{2}\right\rangle & \left\langle p_{1}p_{3}\right\rangle \\ \left\langle p_{3}p_{1}\right\rangle & \left\langle p_{3}p_{2}\right\rangle & \left\langle p_{1}p_{3}\right\rangle \end{array}\right]\approx \left[\begin{array}{ccc} a_{11} & a_{12} & a_{13}\\ a_{21} & a_{22} & a_{23}\\ a_{31} & a_{32} & a_{33} \end{array}\right]$$

The fourth-order stiffness tensors $T_{ijkl}$ according to the fibre orientation tensor P are then obtained for a transversely isotropic material assumption using Equation (9).

(9) $$\begin{array}{c} T_{ijkl}\left(p\right)=B_{1}\left(a_{ijkl}\right)+B_{2}\left(a_{ij}\delta_{kl}+a_{kl}\delta_{ij}\right)\;\\ +B_{3}\left(a_{ik}\delta_{jl}+a_{il}\delta_{jk}+a_{jl}\delta_{ik}+a_{jk}\delta_{il}\right)\\ +B_{4}\left(\delta_{ij}\delta_{kl}\right)+B_{5}\left(\delta_{ik}\delta_{jl}+\delta_{il}\delta_{jk}\right) \end{array}$$

The B-factors are 5 scalar constants related to the independent components of the transversely isotropic elasticity tensor. These are defined as $B_{1}=C_{11}+C_{22}-2C_{12}-4C_{66}$, $B_{2}=C_{12}-C_{23}$, $B_{3}=C_{66}+\frac{1}{2}\left(C_{23}-C_{22}\right)$, $B_{4}=C_{23}$ and $B_{5}=\frac{1}{2}\left(C_{22}-C_{23}\right)$ [40].

The fourth-order orientation tensor $a_{ijkl}$ in Equation (9) is calculated using Equations (10)–(12). The hybrid closure approximation is used to obtain the fourth-order orientation tensor from the second-order orientation tensor, where $f=Aa_{ij}a_{ji}-B$. For orientation in a planar state, A is equal to 2, and B is equal to 1 [40].

(10) $$\begin{array}{c} a_{ijkl}^{LIN}=\frac{1}{7}\left(a_{ij}\delta_{kl}+a_{ik}\delta_{jl}+a_{il}\delta_{jk}+a_{kl}\delta_{ij}+a_{jl}\delta_{ik}+a_{jk}\delta_{il}\right)\\ -\frac{1}{35}\left(a_{ij}\delta_{kl}+a_{ik}\delta_{jl}+a_{il}\delta_{jk}\right) \end{array}$$

(11) $$a_{ijkl}^{QUA}=a_{ij}a_{kl}$$

(12) $$a_{ijkl}^{HYB}=\left(1-f\right)a_{ijkl}^{LIN}+f\;\cdot \;a_{ijkl}^{QUA}$$

### 3.3. Variability of the Fibre Orientation through Thickness

Due to the injection moulding process, there is no uniform fibre orientation through the thickness of the plate [41,42,43,44]. To account for these variations, the injection moulded samples are considered as a multilayer material. In each layer, the orientation distribution is assumed to comply with transverse isotropic material properties. These values are obtained with Autodesk Moldflow simulations using a Midplane mesh consisting of 12 layers (determined by a sensitivity study) [45]. The revised Folgar–Tucker fibre orientation model was selected, introducing standard calculated values for the coefficient of interaction (C_l_) and the thickness moment of interaction coefficient (Dz). This approach gives high accuracy of the predicted fibre orientation in a concentrated suspension, using hybrid closure [46].

**Figure 4:**
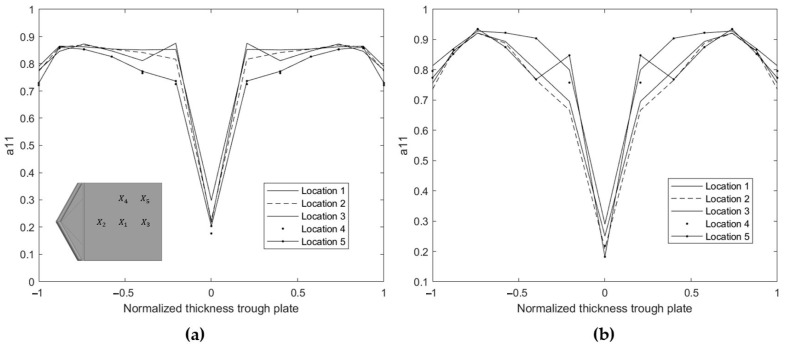
Orientation distribution for a11 through the (normalized) thickness of the plate at different locations, obtained via Moldflow simulations for (**a**) PLA10FF and (**b**) PLA20FF.

The fibre orientation tensor component $a_{11}$ represents the fibre orientation along the flow direction in the plate. In this study, the x-direction is parallel to the flow direction. The numerical results for $a_{11}$ through the thickness of the plates (PLA reinforced with 10- and 20% straw flax fibres) are shown in Figure 4. In this figure, the through-thickness position in the 3 mm thick plates is normalised in the range (–1,1). The $a_{11}$ values are reported for different positions ($X_{1}\to \;X_{5}$) on the plate and visualised in Figure 4. These positions are chosen at specific locations in the plate, covering both the longitudinal and transverse orientations during mechanical testing. Position 1 is at the centre of the plate, positions 2 and 3 are along the orientation of the flow, position 4 is along the perpendicular orientation (y-direction), and position 5 is at an angle of 45° with respect to the x-axis. Results indicate a constant orientation distribution through the thickness over the chosen positions on the plate. Therefore, the fibre orientation tensor (FOT) at the location in the centre of the plate ($X_{1}$) is considered to be the representative distribution over the entire plate. Additionally, the fibre volume fraction affects the final fibre orientation distribution in the plate by changing the viscosity of the SFRT in the molten state [1]. For this reason, a representative FOT distribution is defined per volume fraction of a composite.

Next, the classical laminate theory (CLT) [47,48,49] is applied to obtain the material behaviour of the full SFRT, considering the 12 individual layers of the midplane mesh. The reduced stiffness matrix $\left[K\right]$ is constructed in Equation (16), using Equations (13)–(15). Where k is the number of layers, and z is the height of the bottom of the layer, for which the coordinate system is taken at the centre of the composite. According to the plain stress assumption, a reduced 3 × 3 matrix $\left[Q\right]$ is obtained using the fourth-order stiffness tensor $\left[T\right]$ described in Equation (9). Subscript i refers to each individual layer.

(13) $$\left[A\right]=\;\overset{k}{\underset{i=1}{\sum }}{\left[Q\right]}_{i}\left(z_{i}-z_{i-1}\right)$$

(14) $$\left[B\right]=\frac{1}{2}\overset{k}{\underset{i=1}{\sum }}{\left[Q\right]}_{i}\left(z_{i}^{2}-z_{i-1}^{2}\right)$$

(15) $$\left[D\right]=\frac{1}{3}\overset{k}{\underset{i=1}{\sum }}{\left[Q\right]}_{i}\left(z_{i}^{3}-z_{i-1}^{3}\right)$$

(16) $$\left[K\right]=\left[\begin{array}{cc} A & B\\ B & D \end{array}\right]$$

The 6 × 6 matrix $\left[Q\right]$ of the laminate of 12 layers in Equation (16) is obtained according to Equations (13)–(15). Note that injection moulding allows the presence of a limited amount of out-of-plane oriented fibres. The present model does not allow taking different fibre orientations into consideration. Since the Moldflow simulations indicate values for $a_{33}$ to be smaller than 0.05, this assumption is reasonable.

To predict the stiffness of the composite along these different orientations, only the relevant part of the stiffness matrix $\left[K\right]$ is considered [50,51]. The reduced stiffness matrix $\left[Q^{\prime }\right]$ is rotated for angles Φ in the (1,2) plane. Experimental data for load cases at different angles with respect to the flow direction are compared to the calculated elastic properties under these angles in Equation (17).

(17) $$\left[Q\_angle\right]=\left[T_{1}\right]\left[Q^{\prime }\right]{\left[T_{2}\right]}^{-1}$$

where:

(18) $$\left[T_{1}\right]=\left[\begin{array}{ccc} cos^{2}\Phi & sin^{2}\Phi & 2\cos\Phi \sin\Phi \\ sin^{2}\Phi & cos^{2}\Phi & -2\cos\Phi \sin\Phi \\ -\cos\Phi \sin\Phi & \cos\Phi \sin\Phi & cos^{2}\Phi -sin^{2}\Phi \end{array}\right]$$

(19) $${\left[T_{2}\right]}^{-1}=\left[\begin{array}{ccc} cos^{2}\Phi & sin^{2}\Phi & -\cos\Phi \sin\Phi \\ sin^{2}\Phi & cos^{2}\Phi & \cos\Phi \sin\Phi \\ 2\cos\Phi \sin\Phi & -2\cos\Phi \sin\Phi & cos^{2}\Phi -sin^{2}\Phi \end{array}\right]$$

## 4. Results

### 4.1. Measurement of Fibre Composition

**Figure 5:**
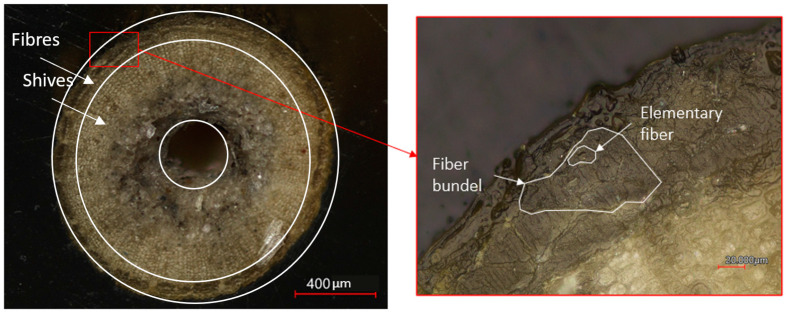
Microscopic image of the straw flax fibre before processing using the Keyence laser microscopy.

Figure 5 presents the interior structure of a flax stem before processing. The elemental fibres are located in the outer layer of the flax stem, while the flax shives are located in the core of the stem. This observation is in line with the literature [23].

**Figure 6:**
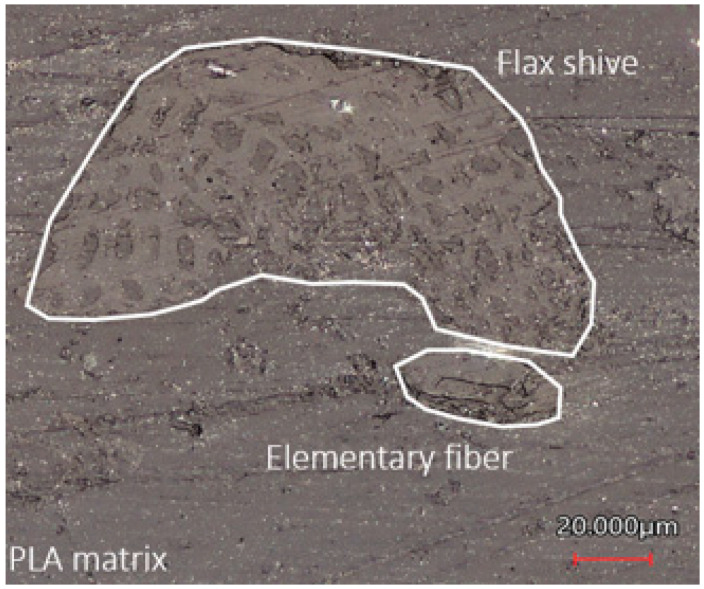
Microscopic image of the reinforcements in the PLA matrix after the production process.

Fibre breakage and splitting of shives and fibres occur due to the high shear forces during processing [24]. This results in flax shives and elementary fibres separately present in the composite. The microscopic image in Figure 6 shows the cross-sectional area of the composite after injection moulding. The impregnation of the PLA matrix within the hollow structure of the flax shives is visible.

The composition of the flax straw used in this work was determined after dissolving the PLA matrix of the injection moulded plates. The results of these measurements are summarised in Table 5 and are consistent with the data in the literature. The shives content is in the range of 70 to 85 V%, depending on the type of flax [20,52].

### 4.2. Measurement of the Fibre Morphology

**Figure 7:**
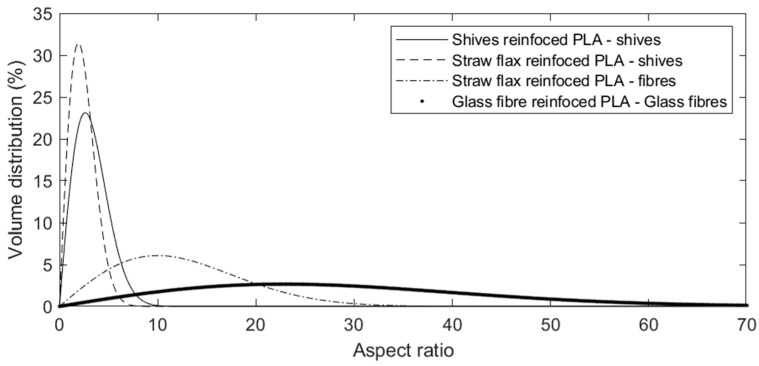
Volume distribution of the aspect ratio of flax shives and fibres after processing the different composite compounds.

The aspect ratio distribution for shives and fibres is shown in Figure 7. Gravimetric separation of shives and fibres (1.5 g/cm^3^ and 1.237 g/cm^3^, respectively [23]) allows the evaluation of their aspect ratio separately. Both number and volume-based distributions of the aspect ratio can be used to analyse the fibre breakage during processing. However, earlier studies indicate that the distribution by volume of the fibres is preferred, as this parameter is most representative of the volume fraction of the reinforcements and, therefore, of the mechanical reinforcement properties [22,24,38]. Therefore, volume-based distribution is reported in this study.

The results summarised in Table 6 indicate a strong reduction in the dimensions of the reinforcements during processing. Average aspect ratios of 2.3 and 9.5 were measured for the flax shives and fibres, respectively. The fibre aspect ratio shows a much wider distribution, compared to the shives (Figure 7). The main difference in size between the two components is shown by the larger average values for the length and width of the flax shives. These act rather as larger particles in the flow.

Furthermore, an average fibre diameter of about 200 µm is measured for the flax fibres. It is known from the literature that the average diameter of an elementary flax fibre is between 40 and 80 µm [50]. This difference between the measured diameter and the literature values suggests that the elementary fibres are not completely separated into individual technical fibres, and that fibre bundles are also present in the compound. This reduces the apparent aspect ratio of the flax fibres, leading to an underestimate of the mechanical stiffness of the composite. To determine the effect of flax fibres on mechanical performance, the average diameter of the elementary fibres is considered. These results also indicate that separation of the fibres from the shives occurs during the compounding and injection moulding step. When technical flax fibres are used as reinforcement, an extra scutching step prior to the processing would be required.

### 4.3. Tensile Properties SFRT Composite

The elastic properties of the SFRT composites obtained after tensile testing are summarised in Table 7. They mainly depend on the type of reinforcement, volume fraction and orientation. Adding 15% of flax shives to the neat PLA material results in a 31% increase of stiffness in the flow direction and a degree of anisotropy of 11%, defined by the relative difference in stiffness in the first and second orientation, written as $\frac{E_{11}-E_{22}}{E_{11}}\cdot 100\%$. Lower pressure values were observed during the melt processing of the compounds for straw flax-reinforced materials. This allowed the production of straw flax-reinforced composites with a volume fraction up to 20%. This is attributed to a smaller increase in viscosity caused by the straw flax, compared to the flax shives. Adding 20% of straw flax of the PLA results in a 58% increase in stiffness and a degree of anisotropy of 21%.

**Figure 8:**
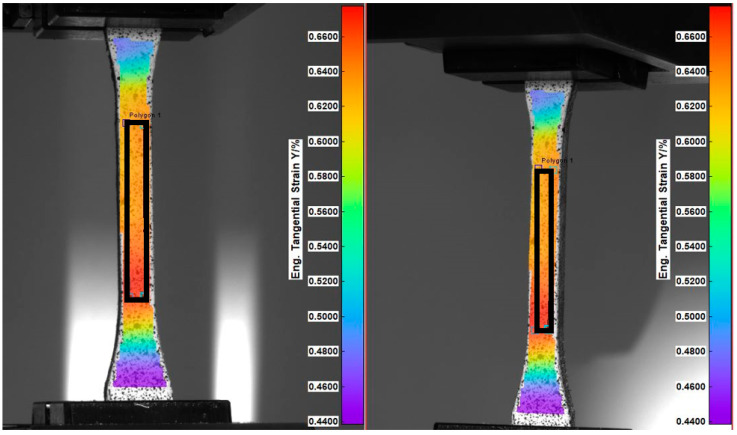
Stereo-vision DIC measurement on tensile bar with defined region for averaging of strain to determine the Poisson’s ratio.

These results show the promising effect of using the whole stem as reinforcement, thus avoiding the extra scutching step beforehand. A variation of less than 5% in the mechanical properties was obtained. This low variation is attributed to well-controlled processing parameters and using fibres and shives from the same batch. The values for the Poisson’s ratios were obtained by taking the ratio of the average strain in the first and second direction over the surface of the tensile bar, as shown in Figure 8.

**Figure 9:**
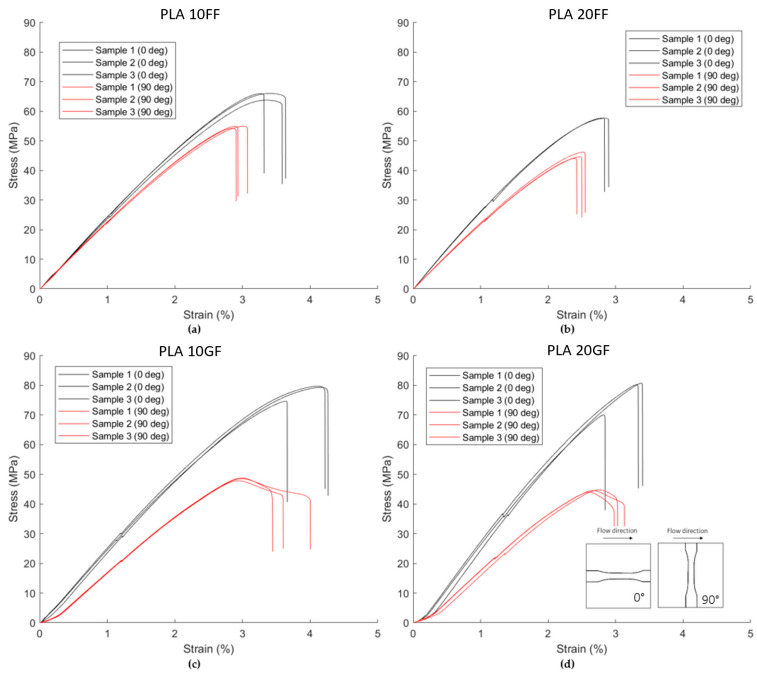
Tensile stress–strain curves in longitudinal and transversal direction according to ISO 527-1BA for (**a**) PLA10FF, (**b**) PLA20FF, (**c**) PLA10GF and (**d**) PLA20GF.

The mechanical properties of glass fibre-reinforced PLA are summarised in Table 8. The stress strain curves are visualized in Figure 9. The increase in tensile stiffness of these composites is slightly higher, compared to the straw flax-reinforced PLA. However, if the density of the reinforcement is considered (2.6 g/cm^3^ [38] and 1.3 g/cm^3^ [23], respectively), the flax-reinforced composite shows a higher specific stiffness than the glass fibre-reinforced composite. A higher aspect ratio of the glass fibres was measured using the same technique as for the straw flax-reinforced composites. This higher aspect ratio is mainly caused by the small diameter of the glass fibres (10 µm), compared to the diameter of the flax fibres. This explains the higher degree of anisotropy. Furthermore, as the flax shives possess an aspect ratio of only 2.1, they rather act as a filler that increases the stiffness in both directions at a lower degree of anisotropy, compared to the fibre-filled composites.

### 4.4. Stiffness Estimation of Flax Shives

**Figure 10:**
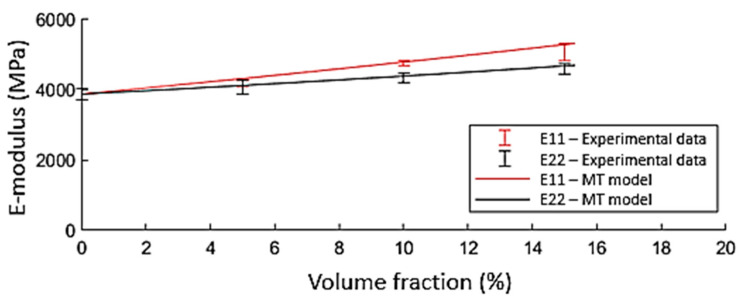
Comparison between the generalised Mori–Tanaka model and the experimental data for flax shives reinforced composites in both the longitudinal and transverse orientation. The presented lines for the Mori–Tanaka model are obtained after optimisation of the cost function.

The literature on the effect of flax shives on a PP matrix indicates that a micromechanics-based model is well suited to determine the elastic properties of the composite [23]. No experimental data on the mechanical properties of flax shives are currently available in the literature. To obtain the stiffness of the flax shives used within this study, a micromechanics-based reverse engineering approach is applied. Due to the low aspect ratio of the flax shives, the effect of the varying orientations trough thickness is negligible. Therefore, a generalised two-phase Mori–Tanaka approach for unidirectional (UD) is used. The error between the predicted tensile modulus C using the generalised Mori–Tanaka homogenisation approach and experimental data for the tensile modulus in both longitudinal and transversal directions at different volume fractions was minimised. The minimisation of the cost function in Equation (20) is obtained by a nonlinear optimisation function in MATLAB. By optimising the cost function, a stiffness of 22.2 GPa was found for the shives. Figure 10 indicates the correlation between the experimental values and the predictive model optimised according to these data.

(20) $$Cost\left(C_{shives}\right)={\;\min}_{i=0\%\;\to \;15\%shives\;}\left(C_{mori-tanaka}\left(i\right)-C_{experimental}\left(i\right)\right)²$$

### 4.5. Fibre Orientation

**Figure 11:**
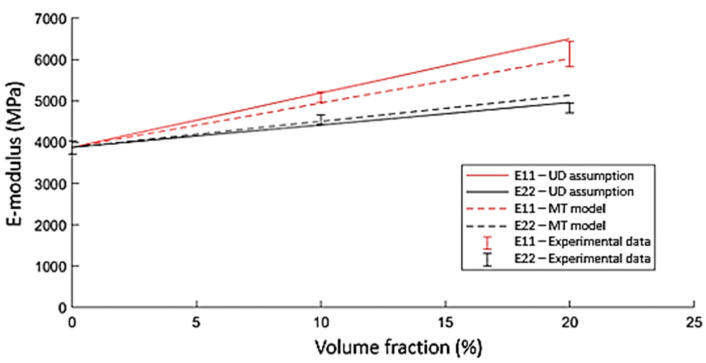
Results of the analytical modelling approach using a UD-based assumption and by considering it to be a laminate consisting of 12 layers with different fibre orientations.

Considering the straw flax reinforced composites, the mechanical properties are initially modelled by assuming a highly aligned SFRT using the proposed generalised Mori–Tanaka approach for a three-phase system. The stiffness of the flax fibre bundles used in this work is reported to be 43.5 GPa in the literature [24]. The other necessary input values were obtained in the experimental part of this study. When the composite is assumed to be a highly aligned SFRT composite material (UD assumption), with no variations in fibre orientation through the thickness, the obtained stiffness data overestimate the experimental data for the longitudinal direction and underestimate the data in the transverse direction (Figure 11). This indicates the expected importance of the through-thickness variation in fibre orientation, caused by the injection moulding process. The use of the multilayer approach (multi-layer setup) leads to a better correspondence of the model to the experimental data. The overestimated stiffness predictions are reduced by lower orientations across the plate thickness.

**Figure 12:**
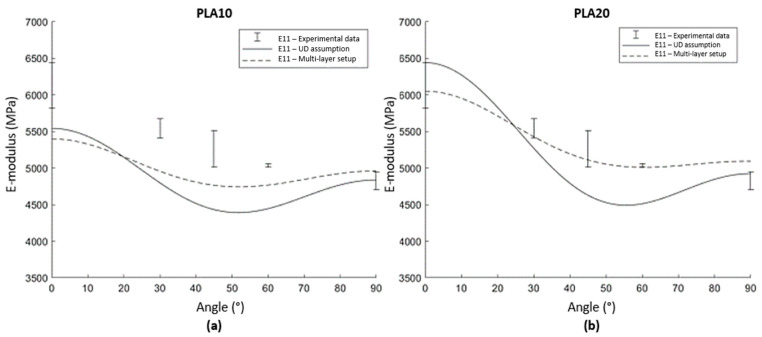
Comparison of the UD modelling approach, the proposed multi-layer approach and experimental data for (**a**) PLA reinforced with 10% straw flax and (**b**) PLA reinforced with 20% straw flax.

The varying fibre orientation through the thickness causes non-negligible deviations in tensile modulus with respect to a unidirectional (UD) orientation of the fibres [46]. The experimental results of tensile stiffness and strength at 0, 30, 45, 60, and 90° are summarised in Table 9. Since the analytical model allows the consideration of any fibre orientation, a comparison of the modelling approach with the experimental data is made. A fibre rotation matrix for the cases between 0° and 90° was used to obtain stiffness data along different directions. The results are shown in Figure 12.

Experimental data are compared with the two modelling approaches. When the UD orientation of the SFRT is assumed, there is a rapid decrease in stiffness as the angle deviates from 0°. When the multilayer approach is used, the stiffness remains at a higher value over a wider range of angles. In this approach, the effect of local fibre orientations through the thickness increases the stiffness in different orientations and thus results in a reduction of this drop in stiffness for off-axis orientations. This highly corresponds to the experimental data, which indicates this effect by showing a slow decrease in stiffness over varying angles with the flow front. Results of this study clearly indicate the importance of taking local orientations into account for short fibre reinforced injection moulded composites, as otherwise the anisotropy over the full composite will be overestimated. The deviation between the UD assumption and the experimental data was shown to be even more pronounced considering the stiffness under various angles with respect to the flow orientation.

## 5. Discussion and Conclusions

Considering the entire flax stem as reinforcement for thermoplastic composites requires less production steps and reduces the cost of time and energy. The potential of flax shives, available as agricultural waste, and straw flax (consisting of fibres and shives) as reinforcing materials in a thermoplastic matrix is investigated in this study. The use of these bio-based reinforcements to produce lightweight and high-performance composites was found to be promising in previous studies that focus on non-renewable feedstock-based matrices such as PP/MAPP [21,22,23]. This work has investigated the applicability of this type of reinforcement in a polylactic acid (PLA) matrix to obtain a fully bio-based material. Moreover, the elastic material properties using a multilayer micromechanical approach were predicted.

Results show that the tensile stiffness of the material increases with increasing volume fraction of the straw flax. These results included an increased degree of anisotropy due to fibre orientation caused by the injection moulding process. For straw flax-reinforced PLA, an increase in tensile strength was observed up to a fibre volume fraction of 10%, after which there was a decrease. Compared to chopped glass fibre-reinforced PLA, the low density of natural fibres leads to a higher specific composite stiffness in the proposed bio-based composite. In addition, the presence of the flax shives in the compound also leads to a lower degree of anisotropy, as they behave more as fillers to increase the stiffness of the matrix.

Furthermore, an analytical modelling approach based on the Mori–Tanaka micromechanical model was set up and validated. By minimising the cost function of flax shives reinforced PLA for different volume fractions, the stiffness of flax shives was estimated via a reverse engineering approach. Assuming that the material is a three-phase system, the dimensions and mechanical properties of both the flax shives and the flax fibres present in the flax stem were considered separately. Injection moulding causes a typical through-thickness variation in fibre orientation, which is taken into account with a multilayer approach, with each layer considered to have its own fibre orientation. This approach resulted in a high correlation of the predicted stiffness with the experimental data in the longitudinal and transverse directions. Assuming complete UD orientation gives an overestimation of the mechanical properties indicating the importance of taking the actual fibre orientation into account, even if the deviation from UD seems rather small. The UD assumption overestimates the drop in stiffness at increasing angle values. The multilayer approach presented in this work was found to be better suited to predict the stiffness of the injection moulded SFRT composites along different orientations.

## Figures and Tables

**Table 1:** Physical properties of the raw fibre materials.

Fibre Type	E-Modulus (GPa)	Tensile Strength (GPa)	Density (g/cm³)	Initial Length (mm)	Initial Diameter (µm)
Glass fibre [38]	70	3.5	2.6	10	10
Flax fibre [23]	45	0.77	1.5	10	±1500

**Table 2:** Compounding parameters.

Parameter	Value
Main feed	150 rpm
Melt pressure	61 bar
Melt temperature	195 °C
Die temperature (∅ 3 mm)	185 °C
Screw configuration (length: 36D) 2D 12–16D 16–20D 28–32D	Feeding PLA Vent Feeding fibres Vacuum

**Table 3:** Injection moulding parameters.

Parameter	Value
Melt temperature	190 °C
Injection volume	30.7 cm^3^
Injection pressure	1065 bar
Injection time	5.8 s
Holding pressure	750 bar
Holding time	15 s
Cooling water temperature	25 °C
Mould temperature	25 °C
Temperature profile in cylinder • T1 • T2 • T3 • T4 • T5 • T6	30 °C 160 °C 170 °C 190 °C 190 °C 190 °C

**Table 4:** Experimental DIC settings and performance (Limess).

Parameter	3D Image Correlation
Sensor type	1/1.2″ CMOS
Resolution	1920 × 1200 px (2.3 Mpx)
Pixel size	5.86 µm × 5.86 µm
Correlation criterion	Universal correlation evaluation
Optimisation residual	0.1349 pixel
Pre-smoothing applied to the images	None
Subset size	17 × 17 pixel
Shape function	Affine
Interpolation function	Bicubic polynomial
Smoothing method	Local regression (kernel size ACSP 05 × 05)

**Table 5:** Composition of the str aw flax fibre.

Component	Weight Fraction	Volume Fraction
Technical fibres	25 wt%	22 V%
Flax shives	75 wt%	78 V%

**Table 6:** Properties of fibre morphology by volume averaging.

Component		Length (µm)	Diameter (µm)	Aspect Ratio (-)
Flax shive-reinforced composite				
Flax shives	Mean. Value St. dev.	1147 ± 109	521 ± 37	2.3
Straw flax-reinforced composite				
Technical fibres	Mean. Value St. dev.	567 ± 215	60 ± 17	9.5
Flax shives	Mean. Value St. dev.	992 ± 212	496 ± 99	2.1
Glass fibre composite				
E-glass	Mean St. dev.	228 ± 140	10 ± 0.1	22.6

**Table 7:** Mechanical properties of SFRT (FS: Flax Shives, FF: Straw Flax Fibres).

Material	E11 Modulus (MPa)	E22 Modulus (MPa)	V12 (-)	Degree of Anisotropy (%)
PLA	3877 (±193)	-	0.35	-
PLA + 5% FS	4186 (±96)	4077 (±239)	0.34	2.6
PLA + 10% FS	4737 (±100)	4337 (±128)	0.34	8.8
PLA + 15% FS	5070 (±307)	4596 (±515)	0.33	11.1
PLA + 5% FF	4284 (±79)	4137 (±135)	0.34	3.5
PLA + 10% FF	5091 (±158)	4563 (±133)	0.33	10.4
PLA + 20% FF	6129 (±58)	4828 (±123)	0.32	21.2

**Table 8:** Specific stiffness (FF: straw Flax Fibres, GF: Glass Fibres).

Material	Young’s Modulus E_11_ (MPa)	$\mathrm{Specific}\;\mathrm{Young}\text{’}s\;\mathrm{Modulus}\;E_{11}/\rho$	Stiffness Ratio (%)
PLA + 10% FF	5091 (±158)	4085	10.4
PLA + 20% FF	6129 (±58)	4895	21.2
PLA + 10% GF	5211 (±103)	3787	27.0
PLA + 20% GF	6475 (±240)	4282	36.1

**Table 9:** Mechanical properties of SFRT under different orientations (FF: straw Flax Fibres).

Orientation	PLA + 10% FF		PLA + 20% FF	
Angle on Flowing Front	E (MPa)	σ (MPa)	E (MPa)	σ (MPa)
0°	5091 (±158)	65.3 (±1.2)	6129 (±58)	58.0 (±0.0)
30°	5051 (±73)	59.3 (±2.8)	5544 (±130)	49.6 (±2.3)
45°	4897 (±243)	51.3 (±0.9)	5263 (±247)	48.3 (±2.3)
60°	4675 (±36)	54.3 (±0.4)	5037 (±23)	44.3 (±2.3)
90°	4546 (±113)	54.4 (±0.4)	4829 (±123)	44.8 (±1.0)

## Data Availability

The data presented in this study are available on request from the corresponding author.
